# How spontaneous brain activity and narcissistic features shape social interaction

**DOI:** 10.1038/s41598-017-10389-9

**Published:** 2017-08-30

**Authors:** Andrea Scalabrini, Zirui Huang, Clara Mucci, Mauro Gianni Perrucci, Antonio Ferretti, Andrea Fossati, Gian Luca Romani, Georg Northoff, Sjoerd J. H. Ebisch

**Affiliations:** 1 Department of Neuroscience, Imaging and Clinical Sciences, G. d’Annunzio University of Chieti-Pescara, Via dei Vestini 33, 66013 Chieti, (CH) Italy; 2 Department of Psychological, Health and Territorial Sciences (DiSPuTer), G. d’Annunzio University of Chieti-Pescara, Via dei Vestini 33, 66013 Chieti, (CH) Italy; 3 Department of Anesthesiology, University of Michigan Medical School CPFRC@Domino Farms Lby M Ste 3100, Ann Arbor, MI 48105-5737 USA; 4 Institute of Advanced Biomedical Technologies (ITAB), G. d’Annunzio University, Via dei Vestini 33, 66013 Chieti, (CH) Italy; 5 Faculty of Psychology, Vita-Salute San Raffaele University, Via Stamira D’Ancona, 20, 20127 Milano, (MI) Italy; 6 The Royal’s Institute of Mental Health Research & University of Ottawa Brain and Mind Research Institute, Centre for Neural Dynamics, Faculty of Medicine, University of Ottawa, Ottawa, 145 Carling Avenue, Rm. 6435, Ottawa, ON K1Z 7K4 Canada

## Abstract

There is an increasing interest in how ongoing spontaneous brain activity and personality provide a predisposition for the processing of environmental demands. It further has been suggested that the brain has an inherent sensitivity to the social environment. Here we tested in healthy volunteers if spontaneous brain activity contributes to a predisposition for social behavior and how this is modulated by narcissistic personality features associated with poor interpersonal functioning. Functional magnetic resonance imaging included a resting state and an experimental paradigm focusing on the anticipation of actively touching an animate (human hand) versus an inanimate target (mannequin hand). The experimental task induced a significant modulation of neural activity in left postcentral gyrus (PostCG), right culmen and, co-varying with narcissistic features, in right anterior insula (AI). Neural activity in anticipation of the animate target significantly correlated with spontaneous activity during the resting state indexed by the Power Law Exponent (PLE) in PostCG and AI. Finally, the correlation between spontaneous and task-induced activity in AI was mediated by narcissistic features. These findings provide novel evidence for a relationship between intrinsic brain activity and social behavior and show how personality could contribute to individual differences in our predisposition to approach the animate world.

## Introduction

Since the early infancy we act in a social environment where we need to distinguish between the animate and the inanimate^1^. This suggests an inherent sensitivity of our brain to the social environment^1, 2^. Conspecifics are intentionally approached as being similar to our selves, with similar inner experiences^3–5^. Correspondingly, social interactions induce neural activity in sensorimotor and affective circuits which allows us to predict and understand others’ sensory experiences^6–8^.

Moreover, our personality is shaped by early interactions with the animate world^1^. The narcissistic personality trait reflects a predisposition for individual differences in social functioning being specifically associated with an inflated sense of self/self-centeredness and dysfunctional interpersonal functioning^8, 9^. On a psychological level, individuals with pathological narcissism may be capable of social processing, but are disengaged from it^11–13^.

However, neither the exact neural mechanisms of the brain’s predisposition for intersubjectivity nor the role of differences in personality features such as narcissism are clear, yet.

Stimulus-induced neural activity has recently been traced to the brain intrinsic or spontaneous activity^14–17^. Raichle^18^ proposed that the brain maintains an intrinsic state of preparedness for anticipating or predisposing the demands placed continuously over time. Indeed, it has been shown that spontaneous activity modulates variability in task-induced activity in sensory cortices^19–23^.

The brain’s spontaneous activity as measured in the resting state (when a participant is awake but not involved in a specific task) shows a complex temporal structure characterized by long-range temporal correlations (LRTCs)^24, 25^. LRTCs are related to a higher time-lagged autocorrelation indicating that the past pattern of a system has a stronger influence on its future dynamics^26, 27^. Concerning functional magnetic resonance imaging (fMRI), previous studies showed that the power law exponent (PLE) can provide a robust and reliable measure of LRTC’s^28^. Higher LRTC’s are indexed by a higher PLE, and imply stronger low-frequency Blood Oxygen Level Dependent (BOLD) signal fluctuations and higher glucose metabolism in the brain^24, 29, 30^.

Initial functional brain states defined in terms of LRTC’s could influence the processing of upcoming stimuli in the environment. Indeed, recent studies demonstrated that the degree of the spontaneous activity’s LRTCs predisposes the neural processing of motor^31^ and sensory (i.e., auditory and visual)^30, 32^ stimuli.

Departing from this background, the present study aims at 1) testing whether task-induced activity during social interaction can be predicted by the spontaneous activity of the brain; 2) to investigate how narcissistic individual differences could mediate the relationship between spontaneous and task induced activity.

In addition to resting state functional magnetic resonance imaging (fMRI), we applied a social interaction fMRI task requiring participants to actively touch an animate (another individual) or inanimate target (a mannequin). Touch plays an incipient role in intersubjectivity^33^, being involved in social interactions already during the earliest stages of life^34, 35^. Furthermore, somatosensory (e.g. postcentral gyrus, inferior parietal gyrus) and affective (e.g. anterior insula, cingulate and orbitofrontal cortices) circuits are involved both in our own experience of touch and in the perception of others being touched^36–38^.

The experimental paradigm focused specifically on the anticipation of animate versus inanimate touch, anticipation referring to the time window preceding the action while already neurally encoding the action including its target^39^. Thus, anticipation reflects a transition phase from a resting state to actual social interaction characterized by the emergence of internally generated behavior without realizing any overt action.

For data analyses, firstly, regions of interest (ROIs) were defined based on the fMRI task (anticipation of animate versus inanimate touch). Secondly, the resting state PLE was calculated for these ROIs and its association with task-induced brain activity was tested. Thirdly, it was investigated if spontaneous and task-induced activity showed neural overlap in their correlation with narcissism. Fourthly, it was tested if the relationship between spontaneous and task-induced activity was modulated by narcissism.

We hypothesized that task induced activity in sensorimotor and affective brain regions in anticipation of touching the animate target, but not the inanimate target, co-varied with spontaneous activity in terms of LRTC’s during a preceding resting state period, whereas narcissistic features could modulate this relationship.

## Methods

### Participants

Thirty-two right-handed male participants (age 21–33; Mean = 25.4; standard deviation = 2.82) were recruited in this study. All participants had normal or corrected-to-normal vision capabilities. None of the participants reported a history of neurological or psychiatric disease, or substance abuse. Written informed consent was obtained from all participants after full explanation of the study procedure, in line with the Declaration of Helsinki. Ethics Committee for Biomedical Research of the provinces of Chieti and Pescara approved the experimental protocol. Participants were paid.

### Data acquisition

fMRI data were acquired by a Philips Achieva MRI scanner at 3 T (See Supplementary Information for more details). All 32 subjects completed the resting state fMRI acquisition. Twenty one out of the 32 participants (age 21–30; Mean = 24.9; standard deviation = 2.45) also completed task fMRI acquisition. In addition to fMRI scanning, all 32 participants completed the Pathological Narcissism Inventory, a 52-item multidimensional self-report measure which was designed specifically to assess both grandiose (NG) and vulnerable (NV) narcissism in the healthy and pathological population^40, 41^. fMRI scanning details and additional information about the PNI can be found in the Supplementary Information.

### Experimental Procedure and Materials

#### Resting state-fMRI

During the two resting state-fMRI runs (6 minutes each), participants were instructed to watch a white fixation cross presented on a black screen, think of nothing in particular and keep their eyes open (they were monitored through a video camera placed in the MRI room).

#### Task-fMRI

The experimental task is based on that used in previous research^42, 43^ and is described in detail in the Supplementary Information. Briefly, during the task fMRI runs (8 runs of 7.8 minutes each), the participant completed a series of touch and no-touch trials. Trial order was randomized. Each trial, either touch or no-touch, started with a visual cue (1000 ms) consisting of a black and white line drawing followed by an attendance signal (red cross for 3000 ms). The drawing indicated the target of the touch (what had to be touched by the participant), that is, an animate (the hand of another volunteer who was standing next to the scanner) or an inanimate target (a mannequin hand). In the no-touch trials (60%), the red cross became black indicating to do noting and wait for the next trial. In the touch trials (40%), the cross became green for 6000 ms and the touch needed to be performed with a brush covert with either velvet (inducing a pleasant sensation when brushing on someone’s skin) or sandpaper (inducing an unpleasant sensation when brushing on someone’s skin).

Since it was not predictable for the participant whether he actually had to perform the touch after the attendance signal, he was forced to be prepared to touch either the animate or the inanimate target after every visual cue. Therefore, the no touch trials allowed to study the anticipation of touching the animate or the inanimate target without the presence of any overt movements of the participant.

Thus, two main conditions could be distinguished: the anticipation of touching an animate target (48 trials) and the anticipation of touching an inanimate target (48 trials).

This task essentially differs from the previous studies for various aspects: 1) by adding the affective component through the valence of the touch (pleasant and unpleasant) the intrinsic link between emotion, self-related processing and social interaction was more specifically considered; 2) a slow event-related fMRI design was used (ITIs = 14000/16000/18000 ms) instead of fast event-related fMRI design; 3) the task was preceded by resting state runs (two independent 6 min-task free fMRI scans acquired before any task) to study the relation between rest and task conditions.

#### Task fMRI analysis

Pre-processing procedures of the fMRI data were implemented in Analysis of Functional NeuroImages software^44^ and are described in the Supplementary Information.

The contrast of principal interest concerned the anticipation of touch an animate target versus the anticipation of touch an inanimate target (is there a difference in the anticipation of touching a human animate hand in contrast to an inanimate mannequin hand?). A whole brain voxelwise paired-sample t-test was performed according to a random effect model to compare neural activity related to the anticipation of touching the animate target and to the anticipation of touching the inanimate target.

Additionally, to test whether individual differences in the neural activity during the task were related to narcissism, whole brain analyses were performed comparing the neural activity during the anticipation of touching the animate target or the inanimate target with baseline, while using the PNI subscales of NG and NV as covariates.

Statistical thresholds for all group statistical maps were set at q < 0.05 after False Discovery Rate (FDR) correction to search for modulations of brain activity by the different targets. The coordinates of the voxel clusters showing statistically significant effects were compared with the Talairach atlas available in AFNI software to label them in terms of anatomically defined regions and Brodmann’s areas (BA).

To explore whether there were statistically significant modulations of BOLD response for the performance of an animate target and an inanimate target touch (touch trials) in the regions of interest (ROIs) modulated by the anticipation of touch, we performed a ROI based analysis. Avoiding circularity in the analysis, individual beta values were extracted from the voxel clusters (ROIs) showing a significant effect regarding the above described whole brain analysis for the anticipation of touch, that is, the no-touch trials. Beta values for each of these independent ROIs were then calculated from the average signal time course of the voxels included in each ROI concerning the performance of touch, that is, the touch trials. A paired-sample t-test was performed on the beta values regarding performance of an animate target touch and an inanimate target touch to establish if there also was a significant difference in neuronal response during touch performance regarding the ROI’s previously associated with the anticipation of touch.

### Resting-State fMRI Analysis: Power Law Exponent (PLE)

Power Law Exponent (PLE) analysis, as a measure of the temporal structure of low-frequency fluctuations^45^, was performed on the resting state fMRI runs^32^. PLE is considered suitable for the measure of scale free dynamics of fMRI data^30^. Comparing different methods for computing fMRI time series complexity, Rubin and colleagues^28^ demonstrated power spectrum based methods such as PLE being among the most robust measures.

Scale-free dynamics are mathematically characterized by a power spectrum following the formula P ∝ 1/f^β^, where P is power, f is frequency, and β is called the “power-law exponent”^45^. After pre-processing, the time course per voxel was normalized to zero mean and unit variance (z-value)^46^. Using methods previously optimized for fMRI^28^, the normalized power spectrum of the fMRI signal was computed for each voxel using AFNI program: 3dPeriodogram. Similar to Welch’s method, the power spectra of the two resting state runs were averaged to reduce noise caused by imperfect and finite data, in exchange for reducing the frequency resolution. The power spectrum of the BOLD signal was further smoothed with a Hamming window (HM) of 7 neighboring frequency bins (HM = 7)^47^. The averaged power spectra across voxels within the a priori ROIs (left PostCG, right culmen and right AI established before on whole brain analysis comparing different targets during the anticipation of touch) were extracted for each participant. The power spectrum was fitted with a power-law function P ∝ 1/f^β^ using a least-square estimation (in a log frequency by log power plot) in the frequency range of 0.01~0.1 Hz^30^. Finally, the power-law exponent, β, of each participant’s ROI was defined as the slope of the linear regression of log-power on log-frequency.

In addition, we performed three different control analysis:

1. To test the goodness of fit for scale invariance in the fMRI signal from particular region of interests we adapted a goodness of fit test developed for testing power-law distributions^48^ and used by other authors in fMRI studies^30, 49^. For each ROI, its time series were extracted for each subject and subjected to PLE analysis for the resting state runs. 1000 time series of fractional Gaussian noise (fGn) with the same length and standard deviation as the original ROI time series were generated. Fractional Gaussian noise is a parsimonious model of stationary scale-free dynamics^50^. Each synthetic fGn time series was subjected to the same PLE analysis as the original resting state data. The p value is defined as the fraction of synthetic time series with standard deviations of residuals from best fit that is larger than the original standard deviations of residuals from best fit of the fMRI time series. The larger the p value, the more plausible the fGn model is for representing the original fMRI time series, and the better the fit of the original data to a scale-free distribution. The hypothesis that the fMRI signal is scale free is plausible if the resulting p‐value is greater than 0.1, otherwise it is ruled out^30, 48, 49^.
2. To confirm the robustness of our frequency domain analysis (PLE) we also independently calculated the Hurst exponent in the time domain with detrended fluctuation analysis (H-DFA)^26, 30^ as a control index and calculated their correlation.
  Specifically, DFA measures the scaling of the root-mean-square fluctuation of the integrated and linearly detrended signals, F(T), as a function of time window size, T. The fluctuation F(T) is of the form F(T) = T^H^, where H is the scaling exponent.
3. Finally, we applied different Hamming Windows (HM = 3, 5, 9, 15) on the PLE calculation to test if the correlation between resting state activity (PLE) and task induced activity could be affected by different smoothing parameters.

### Relationship between resting-state activity and task induced activity

To establish if there was an association between resting state activity (PLE) and task induced activity in the ROIs, we performed Spearman correlation analyses (participant-based) with a 95% confidence interval (CI) based on 1000 bootstrap samples between resting state activity (Beta values) and task-activity (Beta values) for either anticipation of animate target or inanimate target. Bonferroni correction for multiple comparisons was performed on the obtained correlation coefficients, such that only *p* values (before correction) were considered significant below p < 0.05/number of calculated correlations.

In addition to bootstrapping, the correlation was also controlled for all leave-one-out cohorts (N analyses with N-1 participants where each participant is excluded at a time).

### Conjunction analysis: resting state activity and task induced activity with narcissistic features as covariates

It was tested if the relationship between spontaneous activity and task-induced activity is modulated by PNI scores. Firstly, a whole-brain, voxel-wise conjunction analysis was performed to establish whether there was an overlap between brain regions in which task-induced and spontaneous activity both co-varied with narcissistic features. A random effect analysis of the overlap between the two contrasts was based on the minimum statistic compared with the conjunction null^51^. This method controls the false positive error for conjunction inference and tests for common activations by creating the intersection of statistical maps thresholded at a specific alpha rate.

Subsequently, a ROI-based partial correlation analysis was performed using the voxel clusters in which both task-induced activity and PLE correlated with NG or NV as obtained by the conjunction analysis. Specifically, the pair-wise relationships between PNI scores, and task-induced activity (beta scores of task-induced activity during the anticipation of animate touch) and spontaneous activity (beta scores of resting state PLE) in the ROIs were analyzed, while controlling for the third variable.

## Results

### Task fMRI data analysis: anticipation of animate target versus anticipation of inanimate target

**Figure 1:**
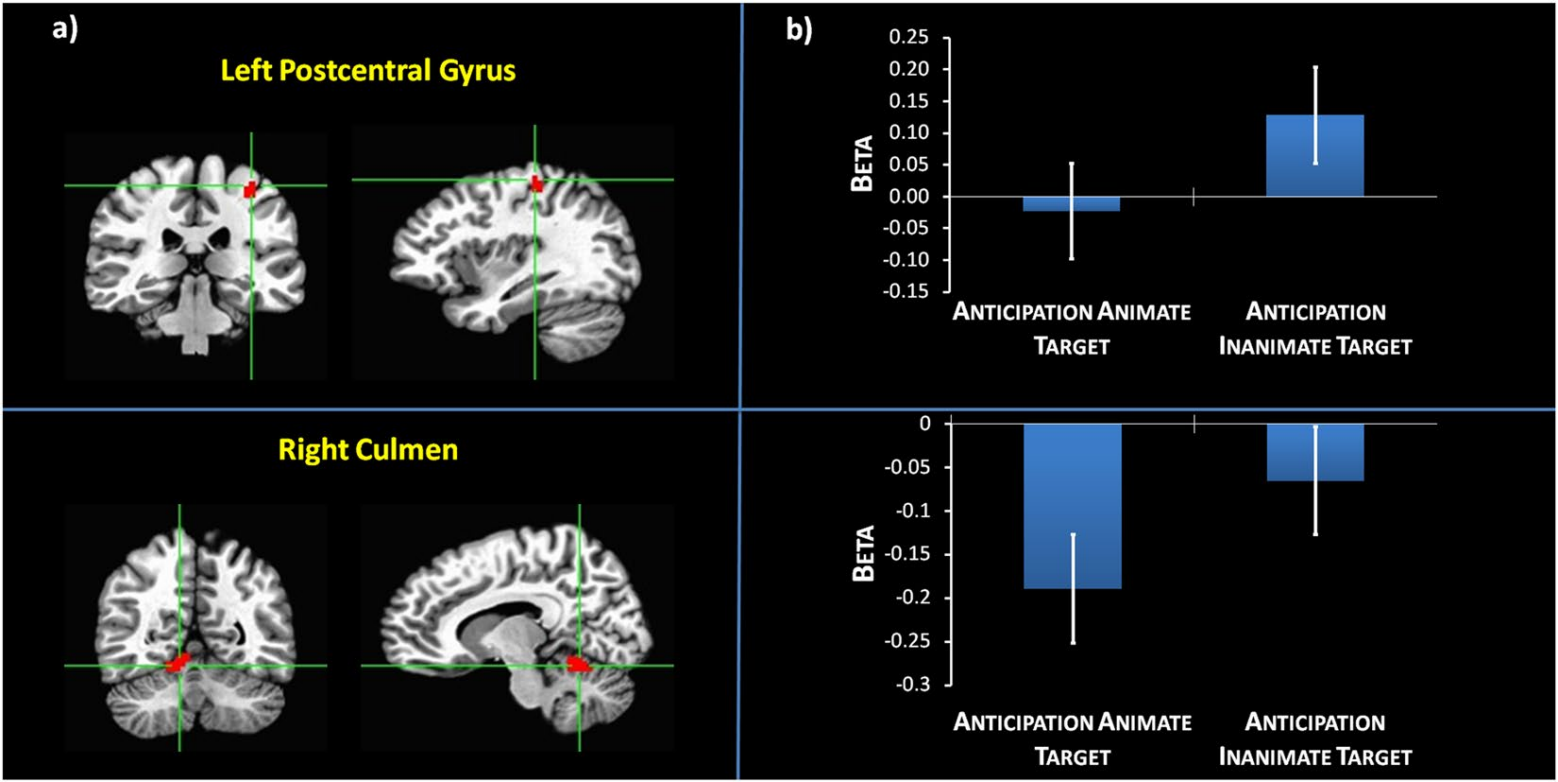
Task-induced activity: (**a**) Group statistical maps of whole brain voxelwise t-test between anticipation of animate target vs. anticipation of inanimate target (t = 3.965; FDR corrected, q = 0.05). (**b**) Graphs of Beta values and Standard Errors extracted from activation clusters depicted in (**a**).

A whole brain voxel-wise paired-sample t-test between anticipation of the animate target and the inanimate target (“no touch trials”) elicited a significant effect in left postcentral gyrus (PostCG) and right culmen (t = 3.965; p = 0.0005; FDR corrected q = 0.05) (Fig. 1, Table 1).

**Table 1:** Brain regions showing a modulation of BOLD response by different experimental conditions and statistical information for the direct contrast between the anticipation of the animate target versus the anticipation of the inanimate target (FDR corrected), for the direct contrast between the anticipation of the animate target versus baseline (FDR corrected) and for the conjunction whole brain analysis between anticipation of animate target vs. baseline with covariate PNI-NG ∩ Resting state PLE vs. baseline with covariate PNI-NG.

Brain region	Broadmann’s Area	C-Mass Coordinates	Cluster Size	t	p
Interaction Contrast (Corresponding to Fig. 1): *Anticipation of the animate target vs. Anticipation of the Inanimate target*					
LH Postcentral Gyrus	3	−29; −33; 52	38	5.116	<0.000005
RH Culmen	—	10; −53; −13	50	5.020	<0.000005
Interaction Contrast (Corresponding to Fig. 2): *Anticipation of the Animate target vs. Baseline with PNI-NG covariate*					
RH Insula	13	35; 17; 8	53	3.828	<0.000001
Interaction Contrast (Corresponding to Fig. 5): *Anticipation of Animate target vs. baseline with covariate PNI-NG* ∩ *Resting state PLE vs. baseline with covariate PNI-NG*					
RH Insula	13	32; 2; 12	44	7.820	<0.00000001

LH = left hemisphere; RH = right hemisphere. C-Mass Coordinates refer to Talairach space. PNI = Pathological Narcissistic Inventory; NG = Narcissistic Grandiosity.

A single subject analysis for the anticipation of animate vs. inanimate target in four randomly selected single participants elicited a significant effect in left PostCG (Supplementary Figure 2).

Examining the graphs, specifically for the anticipation of touching an animate target, we observed no appreciable modulation of BOLD response, compared to baseline, in the left PostCG, whereas a suppression of BOLD response (deactivation) was detected in the right culmen. By contrast, for the anticipation of touching the inanimate target we observed an increased activation, compared to baseline, in the left PostCG, but no appreciable modulation of activity, compared to baseline in the right culmen.

**Figure 2:**
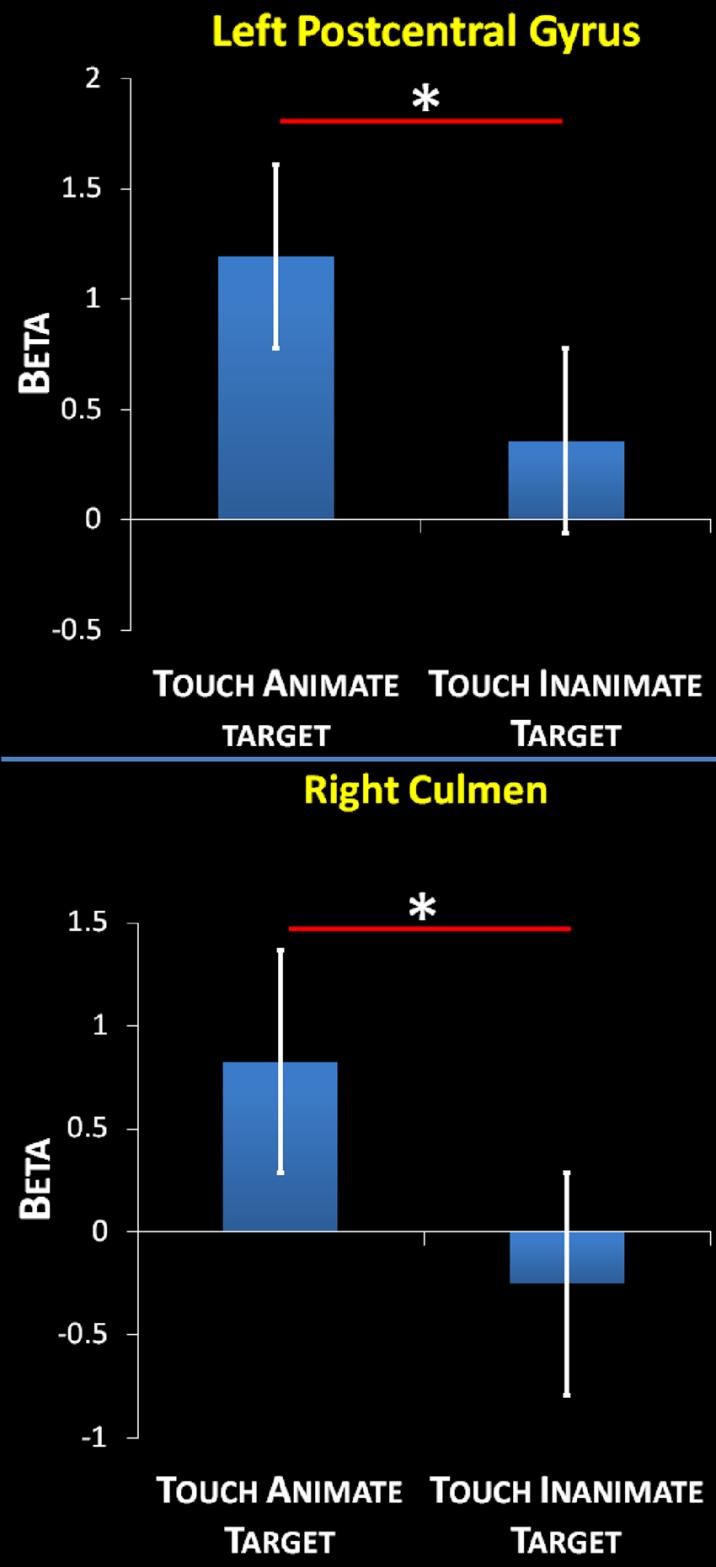
Task-induced activity: ROI based analysis on touch performance in activation clusters obtained by the whole brain voxelwise t-test on the “no touch trials” (touch anticipation; Fig. 1). Bars represent the mean beta value across subject and Standard Error. * indicates p < 0.001.

An exploratory ROI-based analysis yielded the opposite pattern during touch performance (Fig. 2). Specifically, we found a significant difference between the two conditions (touch of an animate target and touch of an inanimate target) both in the left PostCG (p = 0.001) and for the right culmen (p = 0.001). In detail, we observed a greater activation in left PostCG as well as in right culmen during the active touch of the animate target compared to the inanimate target.

**Figure 3:**
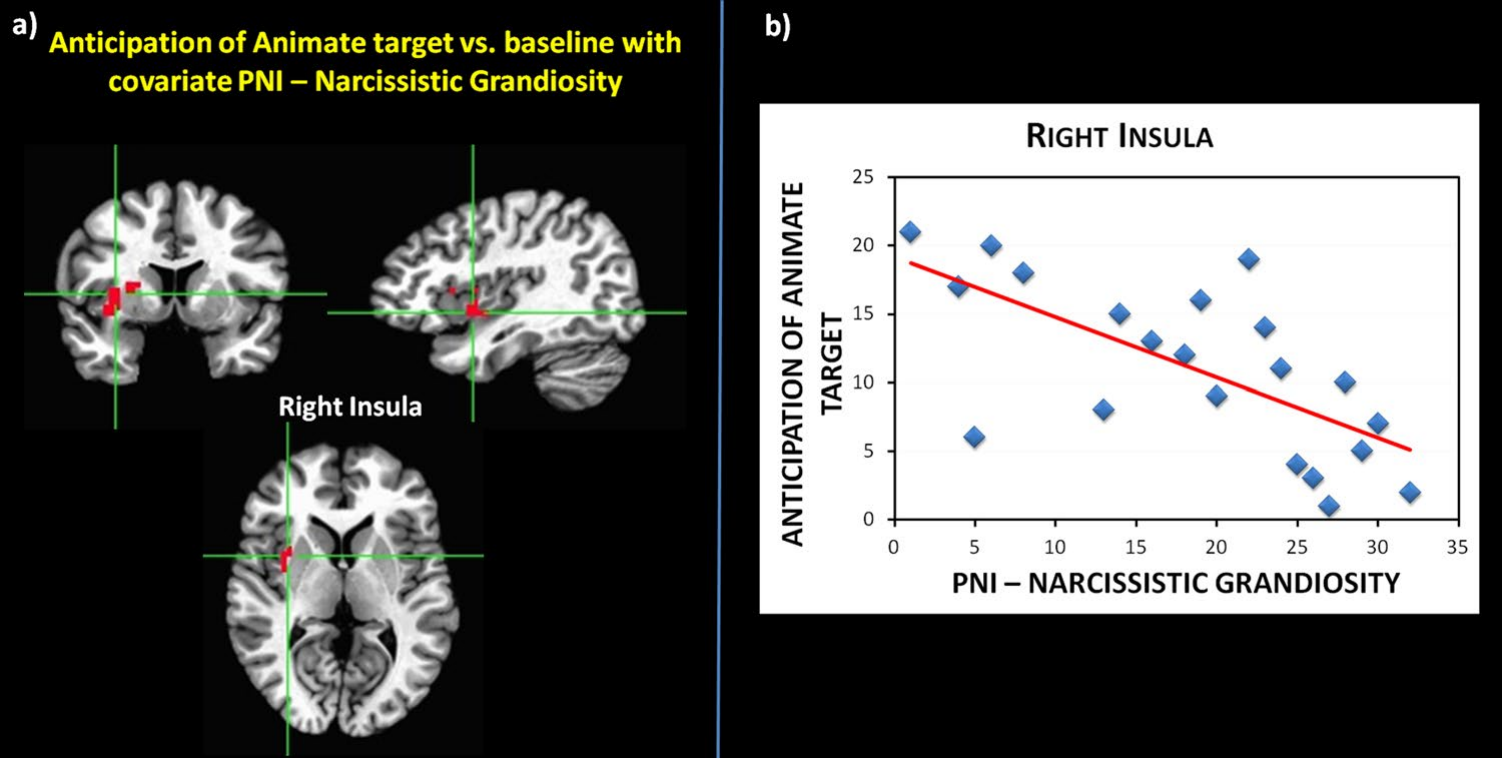
Task-induced activity: Group statistical maps of a whole brain voxelwise t-test between anticipation of animate target vs. baseline with PNI-narcissistic grandiosity (NG) as covariate (t = 3.965; FDR corrected, q = 0.05).

Regarding the co-variance with PNI scores, voxel-wise, whole brain one-sample t-tests on the anticipation of touching the animate target (versus baseline) with NG and NV as covariates elicited a significant effect of NG on BOLD responses in the right anterior insula (AI) (t = 3.828; p = 0.0005; FDR corrected q = 0.05) (Fig. 3), while no significant modulation was reported for NV.

Confirming that the relationship between task-induced activity and PNI scores was specific for the animate target, the same analysis on the anticipation of touching the inanimate target (versus baseline) with NG and NV as covariates yielded no significant effects (t = 3.621; p = 0.001; uncorrected).

### Correlation between PLE and task induced activity

The PLE values across participants (n = 32) in the left PostCG (mean = 0.44; SD = 0.27), in right culmen (mean = 0.37; SD = 0.23) and in right AI (mean = 0.43; SD = 0.19) are in accordance with previous studies^24^.

**Figure 4:**
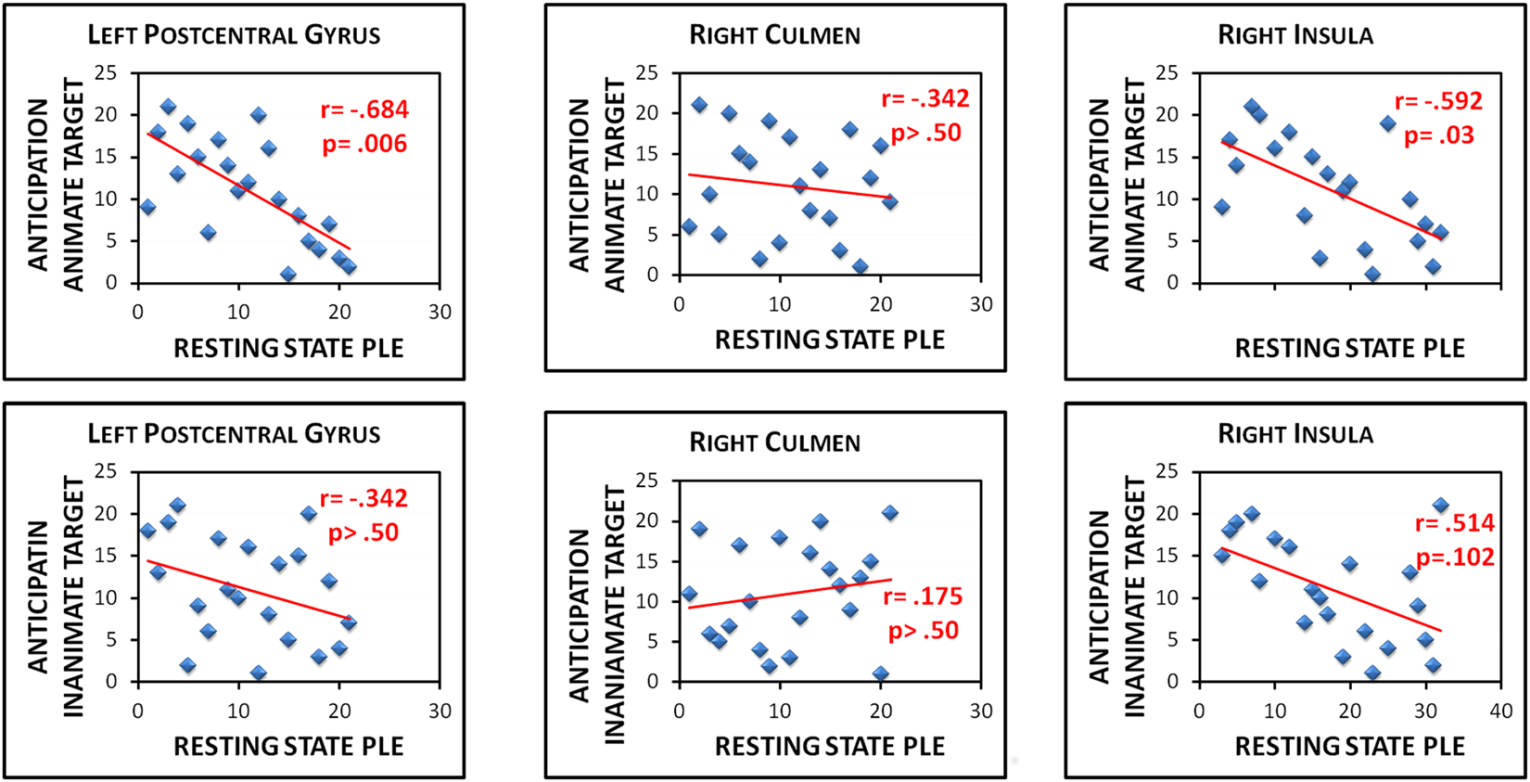
Scatter plots showing predictive power (Spearman correlation) of PLE during the resting state for individual task induced activity in PostCG, culmen and AI ROIs during the anticipation of touching the animate and the inanimate target. *p* values reported in the figure are Bonferroni corrected.

Correlations between resting state PLE and task induced activity were calculated, that is, for task-induced activity in anticipation of the animate and the inanimate touch, in left PostCG, right culmen and right AI (Fig. 4).

A negative and significant correlation between resting state PLE and task induced activity during the anticipation of touch the animate target was observed in left PostCG (r = −0.684, p = 0.006 Bonferroni corrected; 95% CI Lower: −0.874 Upper: −0.332; S.E. = 0.141) and in right AI (r = −0.592, p = 0.03 Bonferroni corrected; 95% CI Lower: −0.822 Upper: −0.217; S.E. = 0.154), but not in right culmen (r = −0.142, p = 0.540 – p = 0.54, uncorrected; 95% CI Lower: −0.600 Upper: 0.283; S.E. = 0.231).

The correlation between PLE and task induced activity was also controlled for all leave-one-out cohorts (N analyses with N-1 participants where each participant is excluded at a time) and this procedure didn’t affect significance of the correlation coefficients for PostCG (min: r = −0.699 p = 0.001; max: r = −0.550, p = 0.012) and for AI (min: r = −0.668 p = 0.001; max: r = −0.543, p = 0.013).

No significant correlation between resting state PLE and task induced activity during the anticipation of touch the inanimate target was observed in left PostCG (r = −0.342, p = 0.130 95% CI Lower: −0.686 Upper: 0.152; S.E. = 0.211), right culmen (r = 0.175, p = 0.447; 95% CI Lower: −0.339 Upper: 0.641; S.E. = 0.244) and right AI (r = −0.514, p = 0.017; 95% CI Lower: −0.867 Upper: 0.019; S.E. = 0.236).

Hotelling-Williams test^52^ was performed to test the equality of two correlation coefficients obtained from the same sample, with the two correlations sharing one variable in common. The test resulted significant for PostCG (z = 2.736; p = 0.006) indicating that the correlation between beta of animate touch anticipation and resting state PLE was significantly stronger than the correlation between beta of inanimate touch anticipation and resting state PLE. The difference was not significant for AI (z = −0.421; p = 0.673) and culmen (z = −1.112; p = 0.266).

### PLE control analyses

(1) Simulating 1000 time series with a stochastic Gaussian process of known long-range temporal dependence, we first showed that the fMRI signal is scale-free by analyzing the goodness of fit indices (left PostCG p = 0.25; right AI p = 0.22; right culmen p = 0.23). Thus, PLE is a suitable measure to quantify the scaling exponent of the fMRI signal.

(2) To further validate the PLE that based on the frequency-domain approach, we applied a time-domain method (DFA) to test for their correlation. As expected, we observed a strong correlation between the two measurements in all the ROIs (for left PostCG, r = 0.722, p = 0.00001; for right culmen, r = 0.804, p = 0.00001; for right AI, r = 0.762, p = 0.00001).

(3) To test the robustness of our results, we applied different smoothing parameters to determine the PLE values, more specifically by varying Hamming window size (HW = 3, 5, 9 and 15). These analyses showed that the correlation of PLE and task induced activity in Post CG and AI for the anticipation of the animate target was not affected by different HMs (Table 2).

**Table 2:** Statistics of the correlation between the Resting state PLE and task induced activity (Beta) in Left Postcentral gyrus, Right Culmen and Right Insula for the Anticipation of the Animate target and Inanimate target.

REST_PLE	LH PostCG		RH Culmen		RH Insula	
	Animate	Inanimate	Animate	Inanimate	Animate	Inanimate
HW = 7	r = −0.684*	r = −0.342	r = −0.142	r = 0.175	r = −0.592*	r = −0.514
	p = 0.001	p = 0.130	p = 0.540	p = 0.447	p = 0.005	p = 0.017
HW = 3	r = −0.662*	r = −0.325	r = −0.152	r = 0.187	r = −0.577*	r = −0.510
	p = 0.001	p = 0.151	p = 0.511	p = 0.417	p = 0.006	p = 0.018
HW = 5	r = −0.677*	r = −0.353	r = −0.152	r = 0.187	r = −0.592*	r = −0.514
	p = 0.001	p = 0.116	p = 0.511	p = 0.417	p = 0.005	p = 0.017
HW = 9	r = −0.652*	r = −0.318	r = −0.152	r = 0.169	r = −0.625*	r = −0.519
	p = 0.001	p = 0.160	p = 0.511	p = 0.464	p = 0.002	p = 0.016
HW = 15	r = −0.694*	r = −0.331	r = −0.205	r = 0.136	r = −0.655*	r = −0.510
	p = 0.000	p = 0.143	p = 0.372	p = 0.556	p = 0.001	p = 0.018

HM = Hamming window size; LH = Left Hemisphere; RH = Right Hemisphere. *p < 0.008 after Bonferroni correction for multiple comparisons.

### Conjunction analysis and partial correlations between Narcissistic Grandiosity, Resting State PLE and task induced activity for the anticipation of the animate target

**Figure 5:**
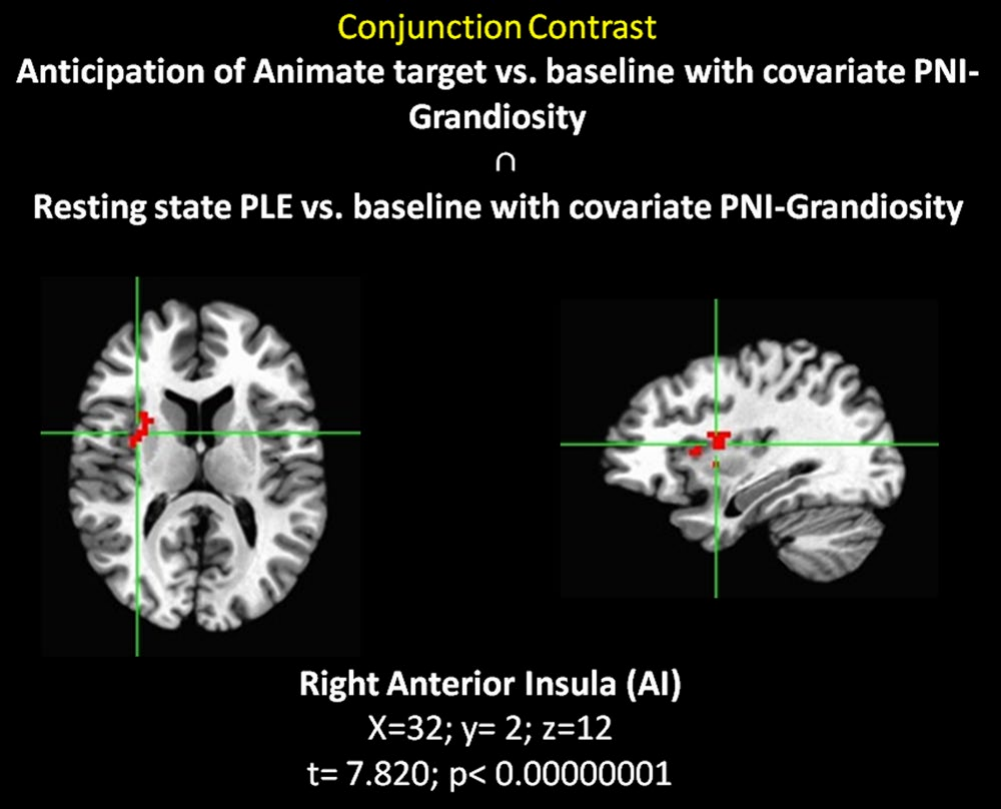
Conjunction contrast between the anticipation of the animate target vs. baseline with covariate Narcissistic Grandiosity and resting state vs. baseline with covariate Narcissistic Grandiosity.

Conjunction analysis showed that right AI activity co-varies with NG both during a resting state (spontaneous activity indexed by PLE) and during task-induced activity (BOLD responses to the anticipation of the animate target) (Fig. 5; t = 7.820; p = 0.00000001). The same analysis using NV as covariates yielded no significant effects (t = 2.750; p = 0.01; uncorrected).

Since spontaneous activity, task-induced activity and NG score all co-varied in AI, partial correlation coefficients were calculated to provide more insight in their interrelationship.

**Figure 6:**
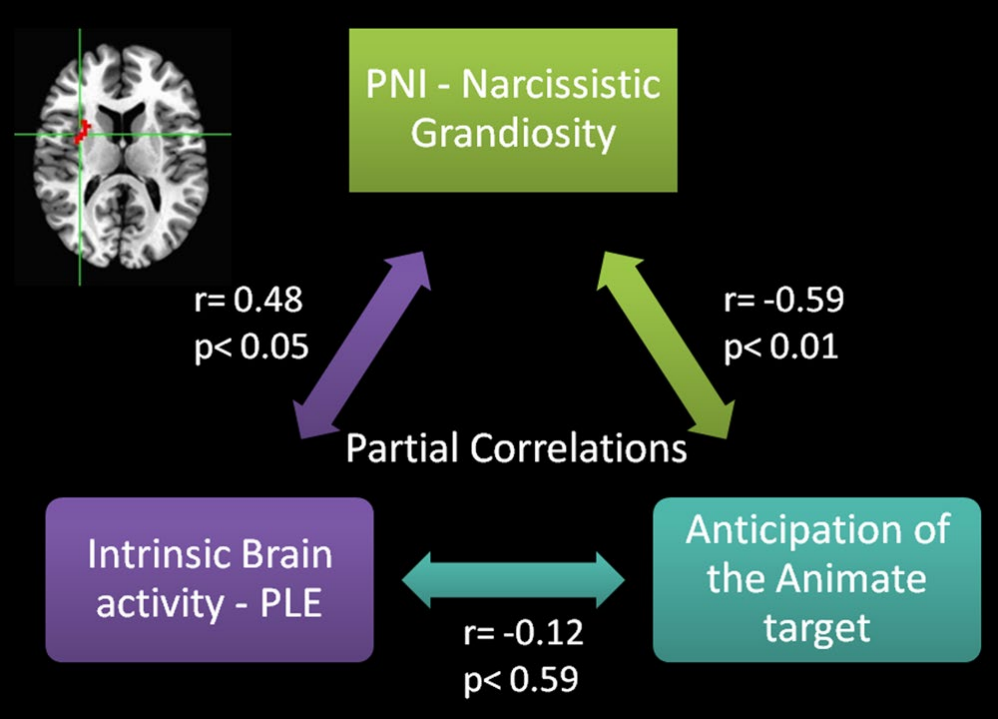
Partial correlations model showing the statistic of each correlation controlling for the effect of the third variable in the model.

Partial correlation yielded a significant positive association between PLE and NG, while controlling for task-induced activity (r = 0.475; p = 0.03; 95% CI Lower: 065 Upper: 0.813; S.E. = 0.189), a significant negative association between NG and task induced activity, while controlling for PLE (r = −0.593; p = 0.006; 95% CI Lower: −0.839 Upper: −0.077; S.E. = 0.203), but no significant association between PLE and task induced activity, while controlling for NG (r = −0.129; p = 0.588; 95% CI Lower: −0.563 Upper: 0.423; S.E. = 0.260) (see Fig. 6).

## Discussion

In the present study, we aimed to investigate whether task activity induced by the anticipation of social behavior (touching an animate target) could be related to the spontaneous activity of the brain during a preceding resting state period, and if this relationship may be mediated by narcissistic traits, particularly NG and NV.

**Figure 7:**
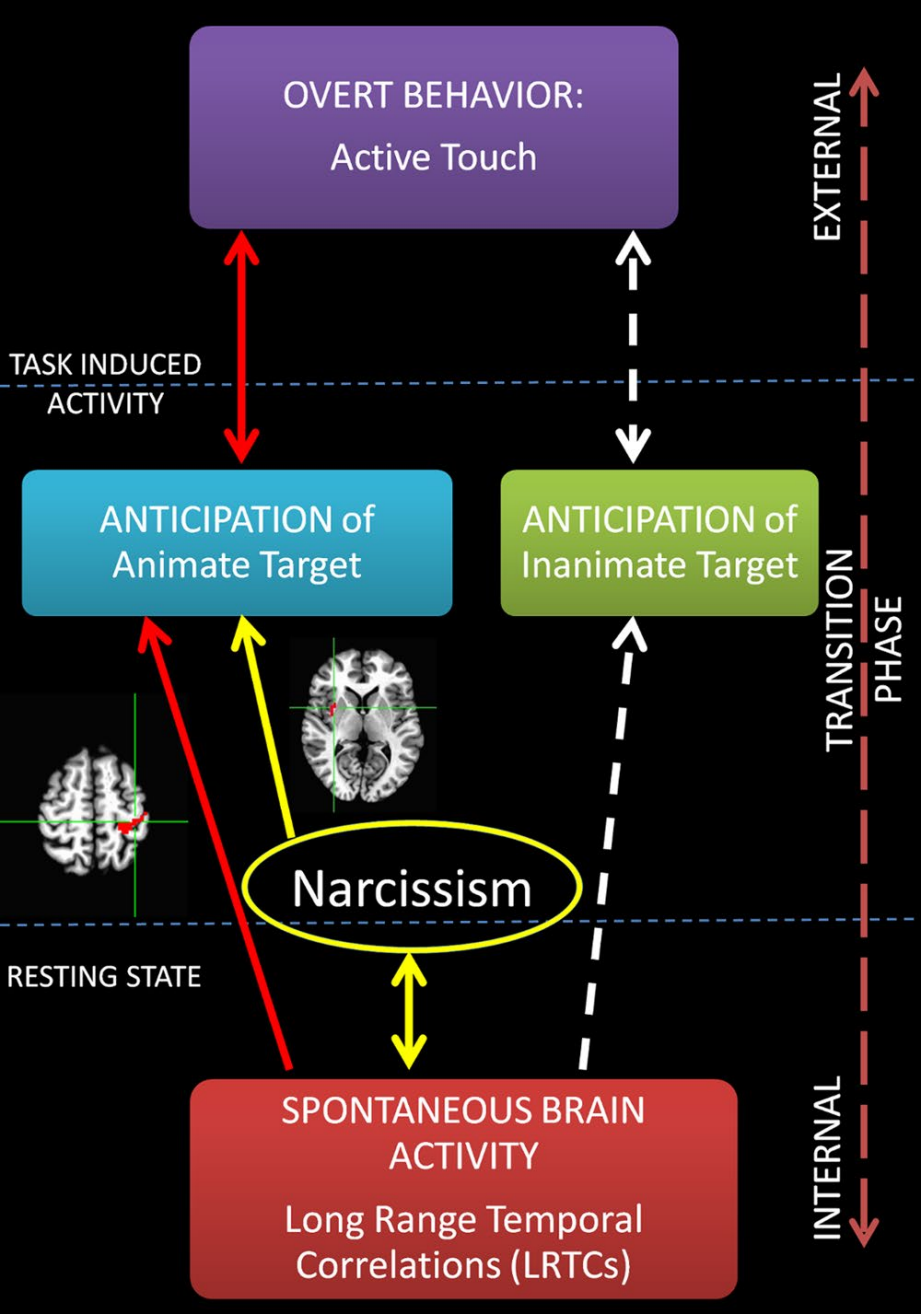
Proposed model of the study visualizing the relationship between intrinsic activity brain activity, task induced activity and the modulation by narcissistic traits. In this model the anticipation is considered as a transitional phase between internal and external where the individual is aware of the external stimuli and is generating internally the behavior without realizing any overt action.

The main results showed that task-induced activity during the anticipation of the animate target (but not of the inanimate target) in left PostCG and right AI negatively correlated with the degree of LRTCs during a preceding resting state: the stronger the PLE in spontaneous activity in left PostCG and AI, the weaker the BOLD response in the same regions for the anticipation of the animate target (see Fig. 7). Interestingly, neural activity in right AI consistently correlated with NG, both during the resting state (PLE) and during task performance (BOLD responses in anticipation of the animate target). Additionally, NG was found to modulate the relationship between spontaneous and task induced activity in the right AI. These data provide, to our knowledge for the first time, evidence for a relationship between intrinsic brain activity and the anticipation of social interaction as well as for how this relationship could be influenced by personality features.

With respect to the rest-task relationship, the detected negative relationship between resting state and task-induced activity is consistent with previous studies showing that the temporal structure of intrinsic brain activity during a resting state can provide a predisposition that shapes our interactions with the world^15, 18, 23, 24, 32, 53, 54^.

Our finding that individuals with stronger LRTC’s in PostCG and AI during a resting state show weaker task-evoked BOLD responses in the same regions during the anticipation of animate interaction, suggests that an individual’s spontaneous brain state defined in terms of LRTC’s might predispose the preparedness for social stimuli in these brain regions.

The result that only the activity induced by the anticipation of the animate target touch, but not of the inanimate target touch, correlates significantly with the temporal structure of the endogenous brain activity in PostCG and in AI is in line with the idea that other individuals are approached as entities with similar inner experiences as our self^2, 3, 6^. Indeed, PostCG and AI have been associated with the perception of one’s own as well as others’ sensations and feelings^33, 36, 55, 56^. Moreover, behavioral results based on “similarity” and “spontaneous social awareness” ratings of touch performance (see Supplementary Figure 1) showed that there is a significant difference in approaching the other (i.e. the animate target) as an entity with similar characteristics of our self, compared to the mannequin.

Hence, the detected relationship between the spontaneous activity and task-induced activity in PostCG and AI supports the hypothesis that others’ bodily experiences might be already internally formulated, as something related to one’s own experiences and that sensory and affective circuits contain a memory trace of it^57^. This seems to be in line with the finding that bodily arousal, linked to psychophysiological states, is associated with spontaneous brain activity during the resting state^58^.

Regarding the relationship between the anticipation and the performance of animate and inanimate touch, PostCG and culmen differentiated between the anticipation of animate and inanimate touch. These regions overlap with those consistently reported in research on sensory anticipation and action prediction^59–61^. According to research on sensorimotor prediction, such sensory activity anticipating the consequences of an action, like touching, may attenuate activity induced by sensory stimuli^62–66^.

Considering this literature, we suggest that a similar predictive mechanism supported by internal simulation may apply to the anticipation of others’ sensations induced by one’s actions^6, 39, 57^. In agreement with this, anticipatory activity in PostCG and culmen showed weaker responses for animate touch anticipation, compared to inanimate touch anticipation, whereas an exploratory ROI-based analysis of BOLD responses during actual touch performance showed the opposite pattern of activity in these regions: increased activity during animate touch performance, compared to the inanimate touch performance. Accordingly, also Gazzola and colleagues^66^ showed increased activity in SI during (passively perceived) affective social touch. Moreover, it is interesting to note that somatosensory activity in SI also supports subjective self-perception induced by tactile stimuli^67^.

Finally, concerning individual levels of narcissism, our data showed that activation specifically for the anticipation of the animate target negatively co-varies with NG in right AI. As evidenced by a conjunction analysis, also spontaneous activity correlated with NG in the same voxels in AI. Partial correlations were performed concerning the relationships between task-induced and spontaneous activity in this AI cluster, and NG. These correlations indicated that the relationship between neural activity in anticipation of the animate target and spontaneous activity during a resting state in AI is no longer significant when controlling for NG, while task-induced activity during the anticipation of the animate target and spontaneous activity during a resting state in AI independently correlate with NG.

On the one hand, the positive correlation between NG and LRTC’s in AI during the resting state period may be interpreted as an increased preoccupation for the self, more specifically the bodily and interoceptive self^68, 69^ during a rest/mind-wandering period^70^. For instance, recent imaging studies showed recruitment of the right anterior insula during tasks focusing on the self ^71, 72^. On the other hand, the negative correlation between NG and task activity elicited by the animate target in AI may be interpreted as a consequent reduced activity regarding other individuals. This hypothesis is also supported by the proposed role of AI as part of the salience network^73^ in constituting a crossroad switch between the internal and the external activity of the brain^74^. Fan and colleagues^58^ specifically showed a decreased deactivation of AI during processing of emotional faces in individual high on narcissistic trait. The authors interpreted their data as indicative of an increased of self-focus and disengagement from empathic processing in narcissistic individuals. The present results extend these findings by showing that higher NG may be related to an increased internal predisposition accompanied by a motivation-based disengagement from social processing^9, 10^.

Thus, these results provide further insight into how personality features may influence brain activity anticipating social interaction. We propose that narcissism could function as a factor mediating between internal processing, related to the self, and external sensory information related to the social world.

Some limitations of the study have to be mentioned. Firstly, we studied the relationship between resting state fMRI and task-induced BOLD responses^32, 75^. It can be argued that this approach is correlational and not directly addresses rest-task interactions^15, 76^. However, because we were interested in how individual spontaneous brain activity patterns could constitute an a priori predisposition for social behavior, intrinsic activity during an independent resting state could be indicated as a more appropriate measure than pre-stimulus activity or background intrinsic activity during task-performance in this context. Nevertheless, further studies have to address direct rest-task interaction integrating these alternative measures that are highly informative for deepening the interaction between endogenous activity and task-induced responses.

Secondly, it can be argued that AI is not a region primarily involved in the discrimination between the animate and the inanimate target. However, AI could be specifically related to grandiose (but not vulnerable) narcissistic features during both the resting state and the anticipation of the animate target. Since NG is characterized by self-serving focus and a motivational based disengagement, it could be speculated that this relation expresses a more general disengagement from the external world in high NG participants. Although this might be not primarily related to the qualification of the target, it possibly is more pronounced for social processing^77^. Further studies would be necessary to clarify this issue more directly.

Thirdly, our sample was not constituted by clinical individuals and further research has to expand this study to clinical samples of pathological narcissism. However, our data lend support to the concept of narcissism as a continuum between healthy and pathological forms reflecting adaptive and maladaptive personality organization, respectively^78, 79^.

In conclusion, our research sheds a novel light on how social task activity can be related to the spontaneous activity of the brain and how this interaction may be modulated by individual personality differences. Future research will need to expand this study to modalities of social interaction other than touch, and to other relevant aspects of personality which may modulate our way to relate with our self and with other individuals.

## Electronic supplementary material

Supplementary Information
