# Supplementary material for: How spontaneous brain activity and narcissistic features shape social interaction
Source: Sci Rep. 2017 Aug 30;7:9986. doi: 10.1038/s41598-017-10389-9 (PMC5577167; doi:10.1038/s41598-017-10389-9)
Supplement: Supplementary file 1 — Supplementary Information [file 41598_2017_10389_MOESM1_ESM.pdf]

SUPPLEMENTARY INFORMATION

How spontaneous brain activity and narcissistic features shape social interaction.

Andrea Scalabrini[1,2]*, Zirui Huang[3], Clara Mucci[2], Mauro Gianni Perrucci[1,4], Antonio Ferretti[1,4], Andrea Fossati[5,] Gian Luca Romani[1,4], Georg Northoff[6], Sjoerd J. H. Ebisch[1,4,]

[1] Department of Neuroscience, Imaging and Clinical Sciences, G. d'Annunzio University of Chieti-Pescara, Via dei Vestini 33, 66013 Chieti (CH), Italy
[2] Department of Psychological, Health and Territorial Sciences (DiSPuTer), G. d'Annunzio University of Chieti-Pescara, Via dei Vestini 33, 66013 Chieti (CH), Italy
[3] Department of Anesthesiology, University of Michigan Medical School CPFRC@Domino Farms Lby M Ste 3100 Ann Arbor MI 48105-5737, USA
[4] Institute of Advanced Biomedical Technologies (ITAB), G. d'Annunzio Foundation, Via dei Vestini 33, 66013 Chieti (CH), Italy
[5] Faculty of Psychology, Vita-Salute San Raffaele University, Via Stamira D'Ancona, 20, 20127 Milano (MI), Italy
[6] The Royal's Institute of Mental Health Research & University of Ottawa Brain and Mind Research Institute, Centre for Neural Dynamics, Faculty of Medicine, University of Ottawa, Ottawa, 145 Carling Avenue, Rm. 6435 Ottawa, ON, K1Z 7K4, Canada
*Correspondence and requests for materials should be addressed to A.S., email: andrea.scalabrini@unich.it)

fMRI data acquisition

For each participant, BOLD contrast functional imaging was performed with a Philips Achieva scanner (Andover, MA) at 3T at the Institute of Advanced Biomedical Technologies, Chieti, Italy. An initial T1-weighted anatomical (3D TFE pulse sequence) was acquired with the following parameters: field of view = 240mm; voxel size = (1mm X 1mm X 1mm); TR 8.1; TE 3.7. Two resting-state fMRI runs (number of fMRI frames/run: 180) and eight task fMRI runs (number of fMRI frames/run: 235) were acquired in a single fMRI session (T2* weighted EPI sequence with TR = 2000 ms; TE = 35 ms; 31 slices; slice thickness = 3.5 mm; in-plane voxelsize = 2.875 x 2.875; field of view = 230 mm; flip angle = 90°).

Additional information on Pathological Narcissism Inventory (PNI)

The concept of narcissism has its roots in psychodynamic theory and it's also central in the diagnosis of personality disorders according to the DSM-5[1].Today, narcissism is considered a personality trait that can be found in healthy subjects and is widely seen as a continuum from healthy self-esteem to severe narcissistic pathology[2].

Narcissism has been conceptualized in two forms: narcissistic grandiosity (NG) is characterized by lack of empathy, arrogant behavior, inflated focus on self and willingness to exploit others individuals[3,4], whereas narcissistic vulnerability (NV) is characterized by susceptibility to self- and emotional dysregulation (e.g., self-esteem, shame, anger, anxiety, envy) when narcissistic needs are not met[4].

Recently, exploratory[5] and confirmatory factor analysis[6] found strong support for a higher-order factor structure that conforms to the theoretical structure of narcissism, with correlated factors representing NG and NV. Higher scores represent higher levels of pathological narcissism. We focused our analysis specifically on the NG and NV scales. The internal consistencies of NG and NV were .86 and .95[7].

fMRI data pre-processing

Pre-processing steps were implemented in Analysis of Functional NeuroImages software[8] including: 1) slice timing correction; 2) rigid body correction/realignment within and across runs. Six head motion parameters, three translational and three rotation parameters, were estimated and frame-wise realignment was performed using AFNI's 3dvolreg command. After the estimated motion parameters were visually inspected, participants with head motion larger than ± 2 mm translation or ± 2.5° rotation were eliminated[9]; 3) co-registration with high-resolution anatomical images; 4) spatial normalization into Talairach stereotactic space; 5) resampling to 3 x 3 x 3 mm^3 voxels; 6) regressing out linear and nonlinear drift (equivalent to a high-pass filtering of 0.0067 Hz), head motion and its temporal derivative, and mean time series from the white matter (WM) and cerebrospinal fluid (CSF) to control for non-neural noise[10]. The WM and CSF masks were eroded by one voxel[11] to minimize partial voluming with gray matter; 7) spatial smoothing with a 8 mm full-width at half-maximum isotropic Gaussian kernel.

Experimental Task-fMRI

During the task-fMRI runs (8 x 7.8 minutes each), a wooden table was placed on the participant's legs. During the experiment, the participant's right hand was resting on an object placed in the center of the table. The object consisted of a brush covert with velvet (inducing a pleasant sensation when brushing on someone's skin) or sandpaper (inducing an unpleasant sensation when brushing on someone's skin). An inanimate hand and an animate target (the hand of another volunteer who was standing next to the scanner) were both placed next to the participant's hand.

To keep the participants naïve about whose hand was placed on the table, they were not introduced to the other person prior to the experiment and it was not be possible for them to see the hand or the individual they touched during the experiment. To avoid systematic effects of the location where the animate and inanimate target were placed, their position was pseudo-randomized throughout the experiment (i.e., on the right and left side of the participant's hand). Before each individual fMRI run, the participant was informed about on which side of his own hand the animate and inanimate target was placed. Behavioral performance accuracy of participant was monitored during the experiment through a video camera placed in the MRI room.

During the task fMRI runs, the participant completed a series of touch and no-touch trials. Trial order was randomized. Each trial, either touch or no-touch, started with a visual cue consisting of a black and white line drawing. The drawing indicated the target of the touch (what had to be touched), that is, the animate or the inanimate target.

The visual cues were presented for a duration of 1000 ms and were always followed by a red fixation cross. After 3000 ms, the red fixation cross could become either green (touch trials; duration = 6000 ms) or black (no touch trials; variable duration = 14000/16000/18000 ms).
Concerning the "touch trials", when the red fixation cross turned green (40% of the trials), the participant was required to rub either the animate target (32 trials) or the inanimate target (32 trials) using the object. When the green fixation cross turned black, the participant had to bring his hand and the object back to the original position on the table.

With respect to the "no touch trials", in case the red fixation cross became black (60% of the trials), the participant was required to keep his hand on the table and to wait for the next cue.

Task fMRI analysis
Trial onsets were defined for a deconvolution analysis (3dDeconvolve in AFNI) using a general linear model approach. Separate predictors were established for the four different types of trials (No-touch animate target or Inanimate target; Touch animate target or inanimate target) and modeled with HRF estimated by the BLOCK option in AFNI. The intertrial interval was used as a baseline period and was not modeled. In addition, the six head motion parameters calculated in the pre-processing steps were included in the model.

The model produces an effect estimate (beta) for each basis function, representing an estimate of the activity amplitude at the corresponding time relative to stimulus onset. In this way, both the shape and the amplitude of the hemodynamic response can be simultaneously estimated.

Behavioral Validation of the task-fMRI stimuli
Prior to the fMRI sessions, behavioral measurements similar to the fMRI task were performed in 74 participants (37 females; mean age 24.4 years, standard deviation = 2.6) not participating in the fMRI experiment. The aim of these measurements was to validate the fMRI task (touching an animate versus inanimate target) as a social task. Participants were asked to follow directions on a screen of a laptop and to touch the hand of another individual (animate target) or the mannequin's hand (inanimate target) with an object covert with velvet (inducing a pleasant sensation when brushing on someone's skin) or sandpaper (inducing an unpleasant sensation when brushing on someone's skin). Different materials were used alternatively as confounders. Participants could only see the hand of the other individual. After the task, participants were asked to answer the questions: "How much did you feel like the other? or How much did you feel like the mannequin?" (translated from Italian) to assess "social awareness"; "How much did you consider the other similar to you? Or How much did you consider the mannequin similar to you?" (translated from Italian) to assess "similarity". To indicate the degree of "social awareness" and "similarity" participant's responses were obtained by a drawing horizontal line on a Visual Analogue Scale (vertical line of 10 cm) ranging from low social awareness to high social awareness and low similarity to high similarity, respectively. Ratings were quantified by measuring the distance in mm between the lower point of the line and the sign of the participant on the scale.

A paired-sample t-test was performed and elicited a significance difference between the animate target and the inanimate target for the constructs of “social awareness” ($t= 13.345$; $p= 0.00001$) and “similarity”($t=9.257$; $p= 0.00001$)

Thus, these behavioral data confirm that the animate target was perceived by the participants as more socially involving as well as similar to the self, compared to the inanimate target.

References

1. American Psychiatric Association, & American Psychiatric Association. DSM-5 Task Force. *Diagnostic and statistical manual of mental disorders: DSM-5™. Arlington, VA: American Psychiatric Publishing* (2013).
2. Watson PJ, Sawrie SM, Greene RL & Arredondo R. Narcissism and depression: MMPI-2 evidence for the continuum hypothesis in clinical samples. *J Pers Assess.* **79(1)**, 85-109 (2002).
3. Pincus, A. L., Ansell, E. B., Pimentel, C. A., Cain, N. M., Wright, A. G., & Levy, K. N. Initial construction and validation of the Pathological Narcissism Inventory. *Psychol Assess.* **21(3)**, 365 (2009).
4. Miller JD, Hoffman BJ, Gaughan ET, Gentile B, Maples J & Keith Campbell W. Grandiose and vulnerable narcissism: A nomological network analysis. *J Pers.* **79(5)**, 1013-1042 (2011).
5. Tritt SM, Ryder AG, Ring AJ & Pincus AL. Pathological narcissism and the depressive temperament. *J Affect Disord.***122(3)**, 280-284 (2010).
6. Wright, A. G., Lukowitsky, M. R., Pincus, A. L., & Conroy, D. E. The higher order factor structure and gender invariance of the Pathological Narcissism Inventory. *Assessment*, ***17*****(4)**, 467-483 (2010).
7. Zeigler-Hill V, Green BA, Arnau RC, Sisemore TB & Myers EM. Trouble ahead, trouble behind: Narcissism and early maladaptive schemas. *J Behav Ther Exp Psychiatry.* **42(1)**, 96-103 (2011).
8. Cox RW. AFNI: software for analysis and visualization of functional magnetic resonance neuroimages. Comput Biomed Res. **29(3)**:162-173 (Jun 1996).
9. Johnstone, T., Ores Walsh, K. S., Greischar, L. L., Alexander, A. L., Fox, A. S., Davidson, R. J., & Oakes, T. R. (2006). Motion correction and the use of motion covariates in multiple‐subject fMRI analysis. *Hum Brain Mapp*. **27(10)**, 779-788.
10. Fox, M. D., Snyder, A. Z., Vincent, J. L., Corbetta, M., Van Essen, D. C., & Raichle, M. E. (2005). The human brain is intrinsically organized into dynamic, anticorrelated functional networks. *Proc Natl Acad Sci U S A*. **102(27)**,9673-9678.
11. Chai XJ, Castañón AN, Öngür D, & Whitfield-Gabrieli S. Anticorrelations in resting state networks without global signal regression. *Neuroimage.* ***59*****(2)**, 1420-1428 (2012).

Supplementary Figure 1.

Paired sample t-test for the construct of spontaneous social awareness for the animate and the inanimate target (t> 13.345; p< 0.0001) and for the construct of similarity for the animate and the inanimate target (t>9.257; p< 0.0001). Graphs represent the mean scores and Standard Error.

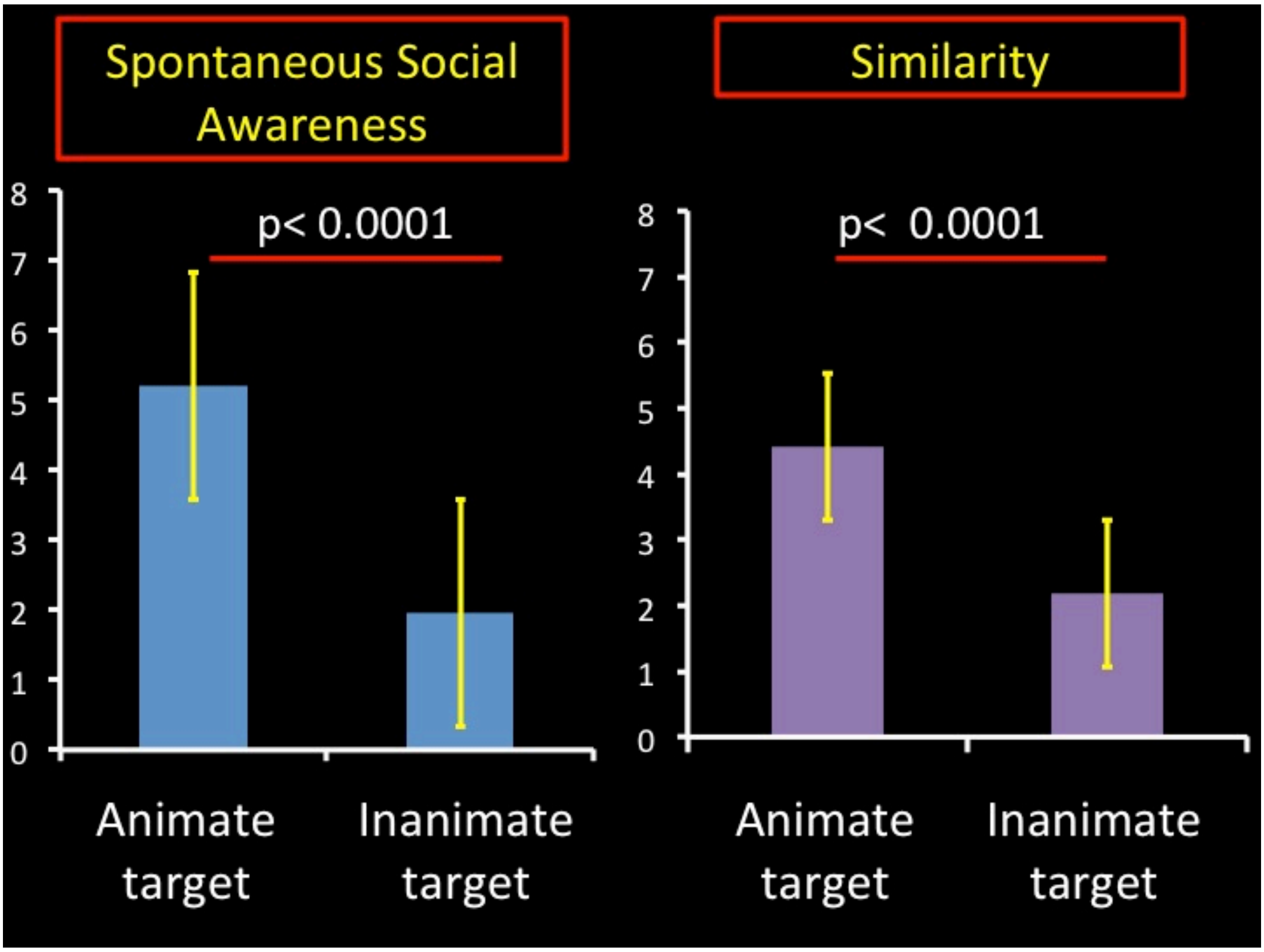

Supplementary Figure 2. The effect of the anticipation of animate vs. inanimate target in four randomly selected single participants. The effect of the significant difference between animate and inanimate target ($p < 0.005$, uncorrected) is shown in left postcentral gyrus (SI).

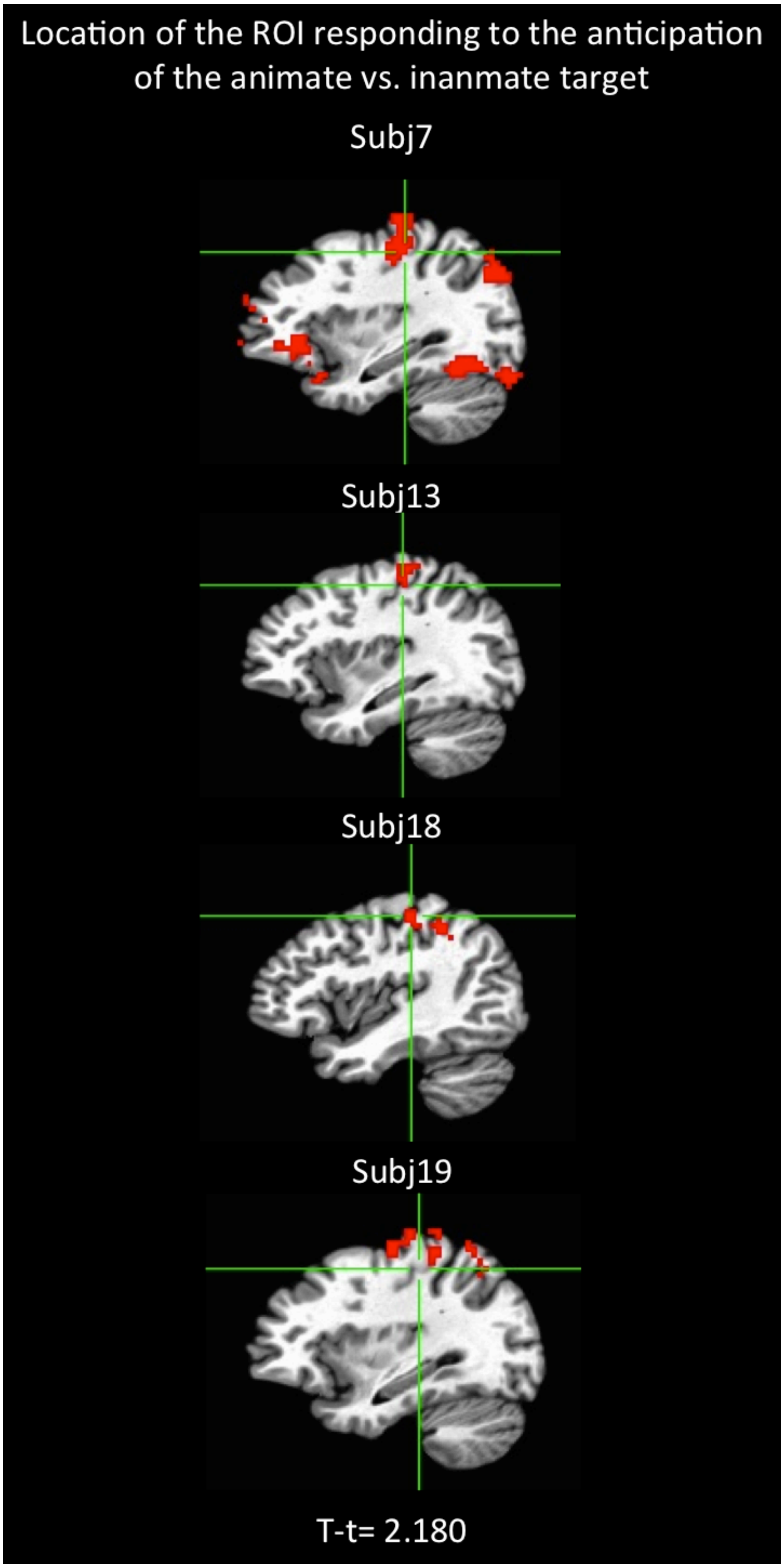
Location of the ROI responding to the anticipation of the animate vs. inanmate target
Subj7
Subj13
Subj18
Subj19
T-t= 2.180
